# SARS‐CoV‐2 spike protein aggregation is triggered by bacterial lipopolysaccharide

**DOI:** 10.1002/1873-3468.14490

**Published:** 2022-09-11

**Authors:** Jitka Petrlova, Firdaus Samsudin, Peter J. Bond, Artur Schmidtchen

**Affiliations:** ^1^ Division of Dermatology and Venereology, Department of Clinical Sciences Lund University Sweden; ^2^ Bioinformatics Institute (BII) Agency for Science, Technology and Research (A*STAR) Singapore City Singapore; ^3^ Department of Biological Sciences National University of Singapore Singapore; ^4^ Department of Biomedical Sciences, Copenhagen Wound Healing Center, Bispebjerg Hospital University of Copenhagen Denmark; ^5^ Dermatology Skåne University Hospital Lund Sweden

**Keywords:** COVID‐19, endotoxins, inflammation, lipopolysaccharide, protein‐aggregation, spike protein

## Abstract

SARS‐CoV‐2 spike (S) protein is crucial for virus invasion in COVID‐19. Here, we showed that lipopolysaccharide (LPS) can trigger S protein aggregation at high doses of LPS and S protein. We demonstrated the formation of S protein aggregates by microscopy analyses, aggregation and gel shift assays. LPS at high levels boosts the formation of S protein aggregates as detected by amytracker and thioflavin T dyes that specifically bind to aggregating proteins. We validated the role of LPS by blocking the formation of aggregates by the endotoxin‐scavenging thrombin‐derived peptide TCP‐25. Aggregation‐prone sequences in S protein are predicted to be nearby LPS binding sites, while molecular simulations showed stable formation of S protein–LPS higher‐order oligomers. Collectively, our results provide evidence of LPS‐induced S protein aggregation.

## Graphical abstract

Bacterial lipopolysaccharide (LPS) plays a central role in SARS‐CoV‐2 spike protein‐induced inflammation. We have previously shown that spike protein binds to LPS. Here, we reveal that at high micromolar concentrations of LPS and spike protein, the spike–LPS complex forms large aggregates. The study complements our previous findings that low subnanomolar concentrations of spike and LPS boost the Toll‐like receptor 4 (TLR4)‐mediated inflammatory response.

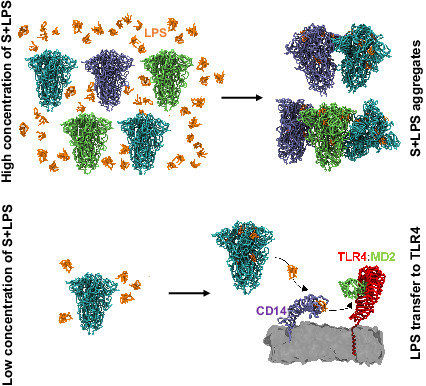

## Abbreviations

**A3D**, Aggrescan3D

**ARDS**, acute respiratory distress syndrome

**BN gel**, Blue Native‐polyacrylamide gel

**CG**, coarse‐grained

**cryo‐EM**, cryogenic electron microscopy

**CTD2**, C‐terminal domain 2

**ECD**, ectodomain

**LPS**, lipopolysaccharide

**MD**, molecular dynamics

**NTD**, N‐terminal domain

**RBD**, receptor binding domain

**RMSD**, root mean square deviation

**S protein**, spike protein

**TCP‐25**, thrombin‐derived peptide

**TEM**, Transmission electron microscopy

**ThT**, Thioflavin T assays

**TLR4**, Toll‐like receptor 4

Spike (S) proteins form projections on the outer surface of enveloped positive‐stranded RNA viruses such as the SARS‐CoV‐2 that cause COVID‐19 [1]. S is a membrane protein made of a large ectodomain (ECD) that comprises the S1 subunit involved in receptor binding and the S2 subunit responsible for membrane fusion. A growing number of studies have shown that S protein directly interacts with bacterial lipopolysaccharide (LPS) [2, 3, 4, 5]. LPS activates the Toll‐like receptor 4 (TLR4) pathway and can trigger a massive release of cytokines, acute phase proteins, and reactive oxygen species. COVID‐19 patients' morbidity and mortality are typically a consequence of severe systemic inflammation and acute respiratory distress syndrome (ARDS) [6, 7]. ARDS is a common systemic inflammatory reaction during pneumonia, sepsis, severe burns, or trauma. Toll‐like receptors, including TLR4 which is stimulated by LPS, are activated during ARDS; hence, the clinical symptoms of patients with ARDS and severe COVID‐19 are similar in the pathophysiology during diseases [8, 9].

In our previous studies, we showed that S protein can bind to LPS *via* multiple sites on both the S1 and S2 subunits, and such interaction with LPS at ultra‐low levels boosts inflammatory reaction *in vitro* and *in vivo* [2, 5]. On the S1 subunit, LPS binds to cryptic pockets on the N‐terminal domain (NTD) and receptor binding domain (RBD), which have been previously shown by cryogenic electron microscopy (cryo‐EM) to bind other hydrophobic molecules [10]. On the S2 subunit, LPS binds to a large groove between the S protein monomers. Interestingly, cryo‐EM structures of S‐protein‐based COVID‐19 vaccine candidates have shown that S protein can also form higher order oligomers, such as a dimer of trimers and a trimer of trimers [11], suggesting potential for S protein aggregation. Indeed, using biophysical and biochemical methods, we previously showed that the addition of S protein beyond the ultra‐low nanomolar concentration to LPS can trigger the formation of high molecular weight complexes [2]. Nevertheless, the effect of LPS on S protein aggregation and the underlying molecular mechanism remains unknown.

We here explored the aggregation of S protein triggered by high doses of LPS. We used electron and fluorescence microscopy to study the size of S protein aggregates before and after LPS‐challenge and showed that LPS induced the formation of larger S protein aggregates compared to S protein alone. Moreover, we validated that the aggregation is dependent on LPS by blocking the LPS‐triggering effect with the anti‐endotoxic peptide TCP‐25. Computational structure‐based aggregation predictions indicated certain regions on the S protein that are aggregation prone, including several loops nearby the LPS binding sites. Finally, coarse‐grained (CG) molecular dynamics (MD) simulations confirmed that higher order S protein oligomers can form stable complexes with LPS.

## Materials and methods

### Proteins and peptides

SARS‐CoV‐2 S protein was produced by ACROBiosystems (Newark, DE, USA). The sequence of SARS‐CoV‐2 S protein contains AA Val 16 – Pro 1213 [Accession # QHD43416.1 (R683A, R685A)]. The thrombin‐derived peptide TCP‐25 (GKYGFYTHVFRLKKWIQKVIDQFGE) (97% purity, acetate salt) was synthesized by Ambiopharm (Madrid, Spain).

### Transmission electron microscopy

S protein (1 μm) was incubated with LPS (50 μm) from *Escherichia coli* or buffer alone for 30 min at 37 °C before the images were taken using transmission electron microscopy (TEM) (Jeol Jem 1230; Jeol, Tokyo, Japan) in combination with negative staining. For the mounted samples, 10 view fields were examined on the grid (pitch 62 μm) from four independent sample preparations. Samples were adsorbed onto carbon‐coated grids (Copper mesh, 400) for 60 s and stained with 7 μL of 2% uranyl acetate for 20 s. The grids were rendered hydrophilic *via* glow discharge at low air pressure. The size of aggregates was analyzed as the mean of gray value/μm^2^ ± SEM by imagej 1.52 k, after all the images were converted to 8‐bit and the threshold was adjusted [12].

### Fluorescence microscopy

We performed staining with Amytracker 680 (Ebba Biotech, Lund, Sweden) to visualize amyloid formation of S protein (1 μm) challenged with LPS (50 μm) for 30 min at 37 °C. The samples (20 μL) were subsequently incubated with 50 μL of Amytracker 680 (1000 × dilution from the stock solution) in the tube for an additional 30 min of incubation at 25 °C. Next, the samples were transferred on (l‐lysine)‐coated glass slides (Thermo Scientific, Braunschweig, Germany), washed and mounted on microscope slides with fluorescent mounting media (Molecular Probes; Life Technologies, Eugene, OR, USA). Ten view fields (1 × 1 mm) were examined from three independent sample preparations using a Zeiss AxioScope (Oberkochen, Germany) A.1 fluorescence microscope (objectives: Zeiss EC Plan‐Neofluar 40×; camera: Zeiss AxioCam MRm; acquisition software: Zeiss Zen 2.6 [blue edition]). The size of aggregates was analyzed as the mean of gray value/μm^2^ ± SEM by imagej 1.52 k, after all the images were converted to 8‐bit and the threshold was adjusted.

### Blue native‐polyacrylamide gel electrophoresis and western blot

Ten microlitre of S protein (1 μm) was mixed with either 10 mm Tris as control, LPS (50 μm), or LPS (50 μm) and TCP‐25 (2 and 10 μm). Samples were incubated for 30 min at 37 °C before mixing with loading buffer (4 × Loading Buffer Native Gel, cat#BN2003; Life Technologies) and subsequently loaded onto 4–16% Bis‐Tris Native Gels (cat#BN1002BOX; Life Technologies). Samples were run in parallel with a marker (Native Marker Unstained Protein Standard, cat#LC0725; Life Technologies) at 150 V for 90 min. The gel was transferred to a 0.2 μm polyvinylidene fluoride (PVDF) membrane (Trans Blot Transfer Pack, cat #1704156, Bio‐Rad, Hercules, CA, USA) *via* a Trans Turbo Blot system (Bio‐Rad). Thereafter, the membrane was de‐stained with 70% ethanol and blocked with 5% milk in 1 × PBS‐Tween (PBS‐T) for 60 min at room temperature. The membrane was incubated with mouse mAb anti‐His tag (cat# MA1‐21315; Thermo Scientific, Rochester, IL, USA), at a concentration of 1 : 1000 dilution in 5% fat‐free milk in PBS‐T, for 1 h at 25 °C. S protein and its high‐molecular weight complexes were detected after adding a secondary HRP conjugated rabbit anti‐mouse polyclonal (cat#P0260; Dako, Santa Clara, CA, USA), which was diluted 1 : 2000 in 1 × PBS‐T complemented with 5% milk and incubated for 60 min at room temperature. The bands were observed upon incubation of the membrane in the developing substrate (Super Signal West Pico PLUS Chemiluminescent Substrate, cat#34580, Thermo Scientific). Signal was acquired using a Chemi‐Doc system (Bio‐Rad). All the experiments were performed at least four times [13].

### Thioflavin T assays

Amyloid formation was determined using the dye Thioflavin T (ThT). ThT (cat# T3516; Sigma, St. Louis, MO, USA) preferentially binds to β‐sheet structures of amyloidogenic proteins/peptides. For examination of the aggregation and blockage of aggregation, we incubated S protein (1 μm) with LPS from *E. coli* (50 μm) or TCP‐25 (2 and 10 μm) in buffer (10 mm Tris, pH 7.4) for 30 min at 37 °C before measurements. Two hundred microliters of the materials were incubated with 100 μm ThT for 15 min in the dark (ThT stock was 1 mm stored in the dark at 4 °C). We measured ThT fluorescence using a VICTOR3 Multilabel Plate Counter spectrofluorometer (PerkinElmer, Santa Clara, CA, USA) at an excitation of 450 nm, with excitation and emission slit widths of 10 nm. The background (10 mm Tris pH 7.4, LPS and TCP‐25) was subtracted from the signal of each sample [14].

### Prediction of S protein aggregation

The structure‐based prediction of protein aggregation webserver, Aggrescan3D (A3D) version 2.0, [15] was used to identify aggregation‐prone regions on the S protein. The cryo‐EM structure of the S protein trimer ECD in the closed state (PDB: 6XR8) [16] was uploaded to the A3D webserver and all three chains were analyzed using default parameters. The aggregation score for each residue was averaged over the three chains and mapped to the cryo‐EM structure. Lipid A molecules were aligned to the binding pockets using structural alignment in PyMOL.

### CG MD simulations of S protein aggregates with lipid A

The atomic structures of S protein trimer aggregates were obtained from the cryo‐EM structures of SARS‐CoV‐2 spike dimer of trimers (PDB: 7JJJ) and trimer of trimers (EMDB: 22355) [11]. These structures represent the ECD of the S protein without the transmembrane and the heptad repeat 2 domains. For the trimer of trimers structure, the atomic structures of individual spike protein trimers were fitted into the EM density map using ucsf chimera. The atomic structures of both dimer of trimers and trimer of trimers were converted to CG representation using the martini 2.2 forcefield with an elastic network applied to maintain the secondary structure of the protein [17]. Parameters for lipid A were taken from previous work [18]. Lipid A molecules were then docked onto the NTD, RBD and the S2 subunit on all chains within the binding pockets described in our previous studies using a structural alignment approach [2, 5]. In total, the spike dimer of trimers was bound to 18 lipid A molecules, whereas the spike trimer of trimers was bound to 27 lipid A molecules. The systems were then solvated with the standard MARTINI water particles and a 0.15 m NaCl salt solution. Steepest descent energy minimisation was performed to move any clashing particles. A 10 ns equilibration simulation was performed for each system, whereby positional restraints with force constants of 1000 kJ mol^−1^ nm^−2^ and 500 kJ mol^−1^ nm^−2^ were applied to the peptide backbone and to the lipid A molecules, respectively. For production simulations, three independent 10 μs simulations were performed for each system using different starting velocities. The temperature was maintained at 310 K and the pressure was kept at 1 atm, respectively, using the velocity‐rescaling thermostat [2] and Parrinello‐Rahman barostat [19]. Coulombic interactions were calculated using the reaction field method, while the van der Waals interactions were truncated using the potential shift Verlet method, both with a short‐range distance cut‐off of 1.1 nm. Both sets of simulations used a 10 fs integration time step.

### Statistical analysis

The graphs of ThT assay, TEM image analysis, fluorescence microscopy image analyses, and blue native‐polyacrylamide (BN) gel image analyses are presented as the mean ± SEM from at least four S protein independent experiments. We assessed differences in these assays using one‐way ANOVA with Dunnett's multiple comparison tests. All data were analyzed using GraphPad Prism (GraphPad Software, Inc., San Diego, CA, USA). Additionally, *P*‐values less than 0.05 were considered to be statistically significant (*P < 0.05, **P < 0.01, and ***P < 0.001).

## Results

### Visualization of S protein‐LPS aggregates

**Fig. 1:**
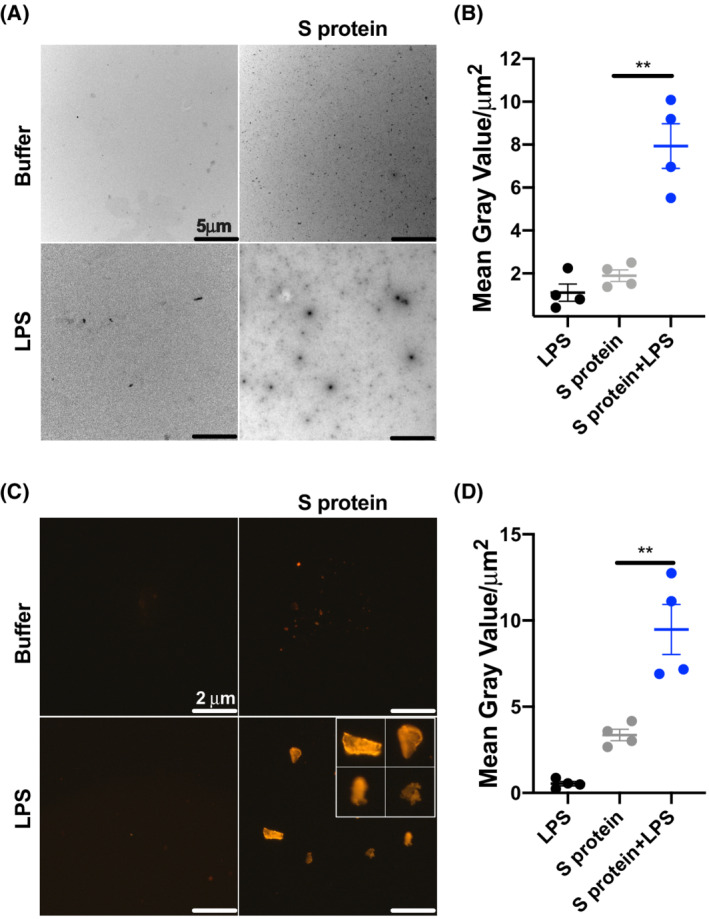
Visualization of S protein aggregation triggered by LPS. (A) TEM‐negative staining revealed rounded aggregating particles of proteins (size from 0.2 to 2 μm) after incubation with LPS from *E. coli*. Smaller and rounded aggregates of proteins were also observed in the untreated samples (0.02–0.2 μm). One representative image from four independent experiments is shown (*n* = 4). The scale bar is 5 μm. (B) Analysis of TEM images was performed to determine the size of particles using imagej 1.52 k. (C) Fluorescent microscopy – Amytracker 680 stain revealed an increase in S protein aggregation (1 μm) when exposed to LPS (50 μm). One representative image of four independent experiments is shown (*n* = 4). The scale bar is 2 μm. The insert represents four aggregates with four times higher magnification. (D) Image analyses of Amytracker 680 signal in S protein‐LPS aggregates. The size of the particles is expressed as the mean of gray value/μm ± SEM. In the graphs, each point represents average measurements of at least 10 pictures per experiment. Statistical analysis was performed using *T*‐test from four independent experiments (*n* = 4). **P ≤ 0.01.

Aggregation of S protein was visualized by negative stain electron microscopy (Fig. 1A). The image analyses revealed that S protein forms larger aggregates upon addition of LPS (Fig. 1B). The results were confirmed by fluorescent microscopy using the dye Amytracker, which specifically binds to β‐sheet structures of amyloidogenic proteins/peptides, yielding a fluorescent signal (Fig. 1C,D).

### TCP‐25 reduces LPS‐induced S protein aggregation

**Fig. 2:**
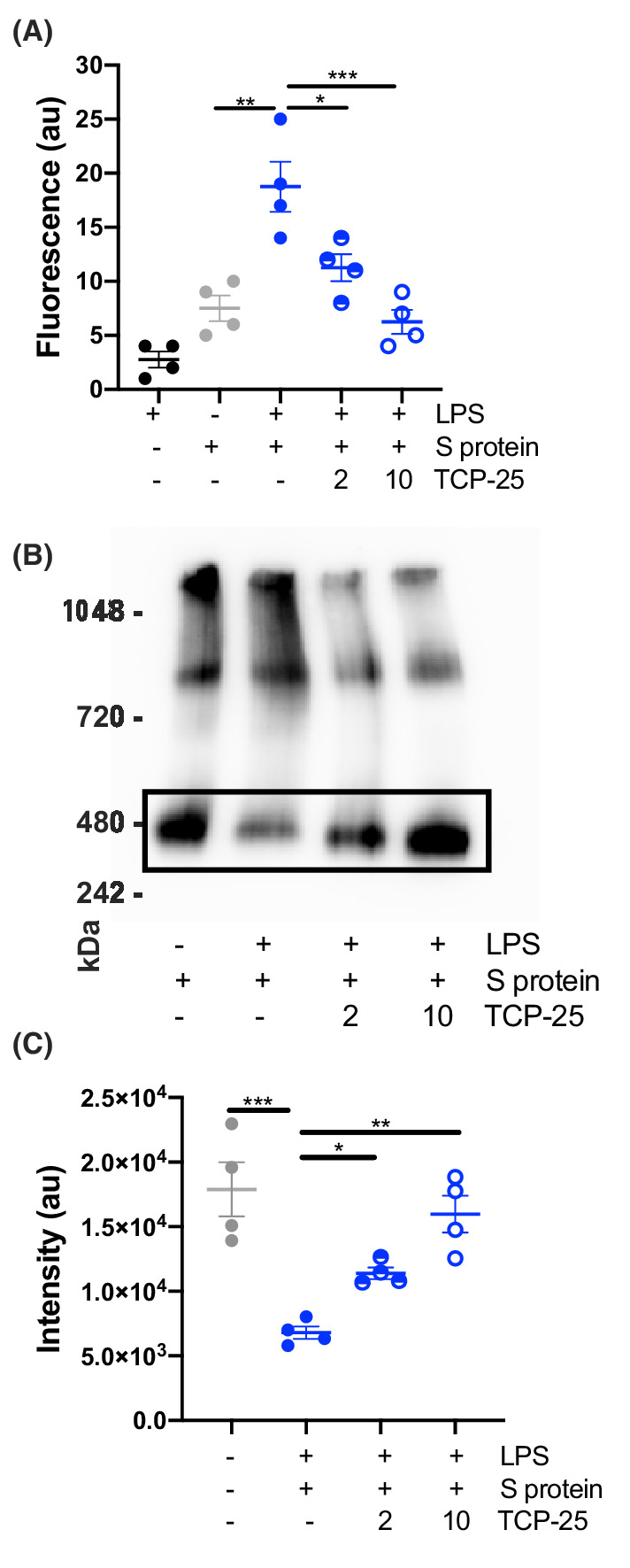
Blocking of S protein aggregation by TCP‐25. (A) ThT aggregation assay confirmed a significant increase of fluorescence in S protein aggregates (1 μm) treated with 50 μm of LPS. The aggregation of the protein was significantly blocked by the addition of 2 and 10 μm of TCP‐25 peptide. (B) BN gel/western blot assays show a significant decrease of monomeric S protein treated with LPS (50 μm) and significant increase after addition of TCP‐25. The protein‐LPS interaction was significantly blocked by both 2 and 10 μm of TCP‐25 peptide. The images represent an example from four independent experiments (*n* = 4). (C) Image analyses of chemiluminescent signal intensity detecting S protein monomer in the presence or absence of LPS and TCP‐25. The fluorescent signal and intensity are expressed as the mean value ± SEM. Statistical analysis was performed using one‐way ANOVA with Dunnett's multiple comparison tests from four independent experiments (*n* = 4). *P ≤ 0.05, **P ≤ 0.01, and ***P ≤ 0.001.

TCP‐25, a peptide derived from the C‐terminus of thrombin binds LPS with high affinity [18]. To demonstrate that the aggregation of S protein is LPS‐dependent an excess of TCP‐25 was added to the mixture. Thioflavin T1 is a fluorescent dye that binds specifically to amyloidogenic proteins exhibiting β‐sheet structural features. The fluorescent signal of S protein significantly increased upon LPS‐treatment, whereas TCP‐25 (at 2 and 10 μm) significantly blocked LPS‐mediated S protein aggregation (Fig. 2A).

Furthermore, we validated aggregation of S protein treated by LPS using native blue gel/western blot analyses. We ran electrophoreses under non‐denaturing conditions to detect the formation of high molecular complexes of S protein in the presence of LPS. We detected a significant decrease in the monomeric (non‐aggregated) form of S protein when S protein was mixed with LPS. This monomeric band increased again when we added TCP‐25 (2 and 10 μm) to the mixture of S protein and LPS (Fig. 2B). Image analyses revealed significant changes of the S protein monomer subjected to LPS alone or in combination with TCP‐25, respectively (Fig. 2C). Taken together, the results showed that LPS induces S protein aggregation leading to amyloid formation.

### Aggregation prone regions on S protein nearby LPS binding sites

**Fig. 3:**
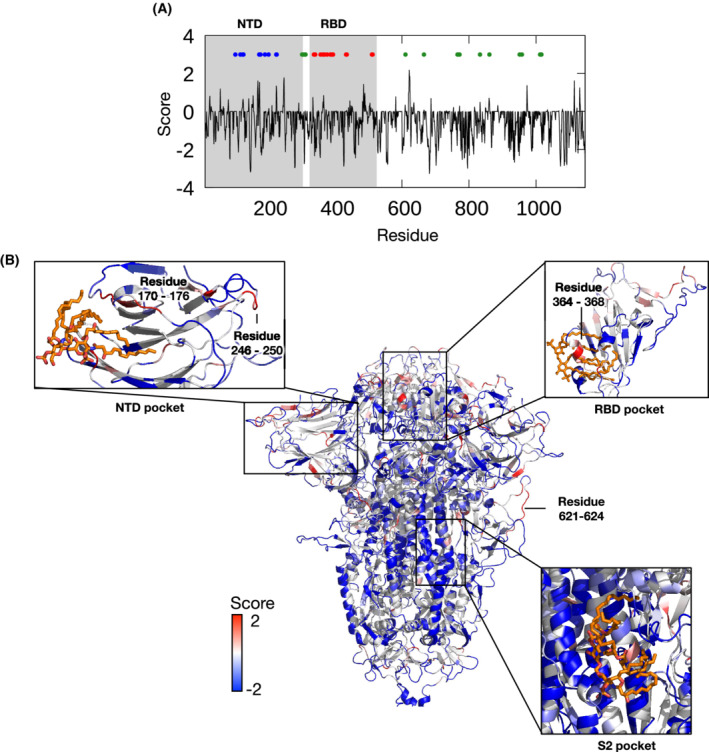
Aggregation propensity of SARS‐CoV‐2 S protein. (A) Aggregation score predicted by the A3D 2.0 webserver for the S protein trimer. Shaded areas indicate the positions of the NTD and RBD. Dotted circles indicate residues involved in LPS binding as determined by our previous studies [2, 5] (blue, NTD pocket; red, RBD pocket; green, S2 pocket). (B) the S protein trimer colored by aggregation score from blue (least aggregation‐prone) to red (most aggregation‐prone). Enlarged images show LPS binding sites with lipid A moiety shown in orange stick representation. Regions with high positive scores are labeled.

To understand the molecular mechanism of S protein aggregation in the presence of LPS, we first investigated the potential regions on the S protein that are prone to aggregation using the A3D webserver [15]. The aggregation propensity score for each S protein residue, whereby a positive value suggests a high propensity for aggregation and a negative value suggests a low propensity for aggregation, is shown in Fig. 3A. There are several stretches of residues that have positive scores and thus may contribute toward S protein aggregation. These include loop 246–250 on the NTD and loop 621–624 nearby the C‐terminal domain 2 (CTD2). In cryo‐EM structures of SARS‐CoV‐2 S‐protein‐based vaccine candidate, the S protein forms higher‐order complexes *via* interactions between these two loop regions [11]. The loop 621–624 is also a part of a potentially druggable cryptic pocket that we previously identified and lies nearby several mutations found in SARS‐CoV‐2 variants [20]. Interestingly, this loop is adjacent to peptide 601–620, which forms amyloid fibrils when the S protein is co‐incubated with neutrophil elastase *in vitro* [21]. Additionally, two LPS binding pockets, i.e., the NTD and RBD pockets [5], are found in close proximity to several regions with positive aggregation propensity scores (Fig. 3B). Loop 170–176 is a part of the upper edge of the LPS binding site on the NTD. This loop and surrounding glycan molecules have been shown to mediate the opening and closing of the LPS binding pocket [20]. In the RBD, residues 364–368 form a “gating” helix that moves away to allow LPS binding [5]. A high affinity LPS binding to the RBD pocket is dependent on the helical secondary structure of this region [5]. As both aggregation‐prone regions are nearby LPS binding sites and play crucial roles in S protein‐LPS interaction, it is conceivable that LPS binding could modulate S protein aggregation *via* these residues.

### S protein‐LPS forms stable higher order oligomers

**Fig. 4:**
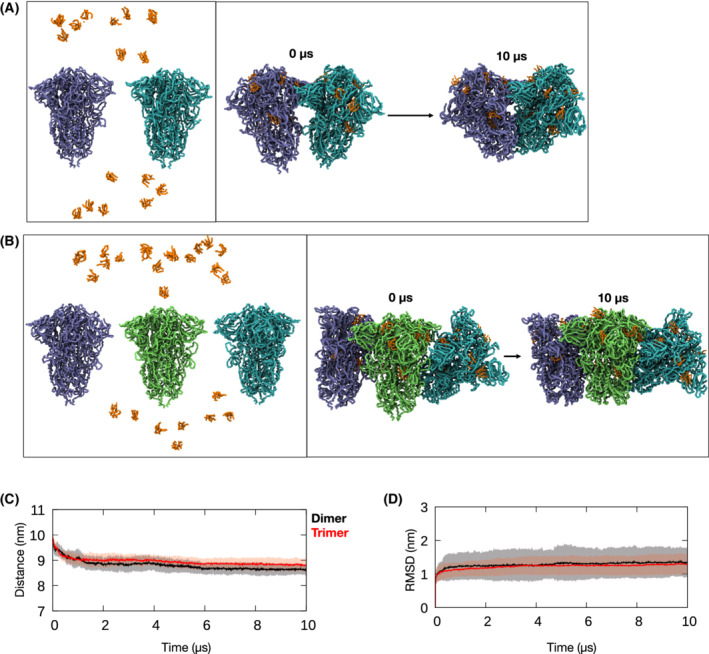
CG MD simulations of S protein aggregates bound to lipid A. (A) CG MD simulations of two S protein trimers (cyan and purple) and 18 lipid A (orange) were performed (left figure). The starting structure of the S protein dimer of trimers was taken from PDB: 7JJJ. The initial and final snapshots from a 10 μs simulation are shown on the right. Each monomeric unit of the S protein trimer is bound to 3 lipid A molecules at the NTD, RBD and S2 pockets as previously described [2, 5]. (B) Similar simulations were performed for three S protein trimers (cyan, green, and purple) and 27 lipid A molecules (left figure). The starting structure of the S protein trimer of trimers was taken from EMDB: 22355. The initial and final snapshots from a 10 μs simulation are shown on the right. (C) the distance between the centers of mass of the S protein trimers in simulations of the dimer of trimers (black) and trimer of trimers (red). Average from three independent simulations are shown as thick lines and the standard deviations are shown as shaded areas. (D) RMSD of the lipid A molecules from simulations of the S protein dimer of trimers (black) and trimer of trimers (red). The RMSD values are averaged over all lipid A molecules and the three simulations performed. Average values shown as thick lines and the shaded area depict standard deviation.

Next, to investigate the stability of S protein aggregates in the presence of LPS, we performed CG MD simulations of S protein aggregates in the presence of lipid A, the core lipid component of LPS that encompasses the primary stimulatory activity for TLR4. To date, there are two cryo‐EM structures of higher order S protein oligomers, i.e., an S protein dimer of trimers and an S protein trimer of trimers from a SARS‐CoV‐2 vaccine candidate [11]. Each of the LPS binding sites were loaded with lipid A according to our previous structural studies [2, 5], and the S protein‐lipid A complex systems were simulated in triplicate for 10 μs (details in Materials and Methods). Representative snapshots from the beginning and end of the simulations are shown in Fig. 4A,B for the S protein dimer of trimers and S protein trimer of trimers, respectively. Overall, the higher‐order S protein‐lipid A complexes remained stably bound in both sets of simulations; in all cases, the mean distance between the S protein trimeric units reached a plateau after the first 2 μs, and in fact gradually reduced by ~ 1 nm on average, compared to the initial structures (Fig. 4C).

We next sought to confirm that all lipid A molecules remained bound to the S protein pockets. This was assessed by measuring the root mean square deviation (RMSD) for each lipid A molecule, after least‐squares fitting to the initial structure of the respective S protein complex. As shown in Fig. 4D, the magnitude of the RMSDs averaged over all bound lipid A molecules remained stable at ~ 1.5 nm throughout the entire simulations of both the S protein dimer of trimers and trimer of trimers, consistent with no detachment of lipids from their binding pockets. Our simulations, therefore, demonstrate that the S protein‐lipid A complex can in principle form stable higher order oligomers or aggregates, consistent with the experiments described above. A limitation of the CG approach is that the elastic network model applied to the protein prevents large‐scale changes in the secondary structure of the protein. Thus, we would not be able to observe any potential conformational changes elicited by lipid A binding that may promote the formation of S protein aggregates. Due to the large size of the systems, simulations at the atomic level were not considered in this work. Nevertheless, our simulations provide a proof of principle at the molecular level for S protein aggregation in the presence of LPS.

## Discussion

Here we demonstrate that LPS induces aggregation of SARS‐CoV‐2 S protein, leading to formation of amyloid structures. In the presence of LPS, S proteins assembled into aggregates that are significantly larger than the ones formed by S protein alone. The LPS‐sequestering TCP‐25 reversed this effect, thus confirming the role of LPS in S protein aggregation. Predicted aggregation prone regions on the S protein overlap with LPS binding sites on the S1 subunit. Simulations of higher order S protein oligomers bound to lipid A showed stable complexes, further corroborating that S proteins can form aggregates with LPS molecules.

Recently, it was shown that several peptides generated upon proteolysis of the S protein by neutrophil elastase formed aggregates, of which three (peptide 192–211, 601–620, 1166–1185) fulfilled the amyloid fibril criteria [21]. It is therefore interesting that we predicted that loop 621–624 in the CTD2 domain, which is adjacent to one of these peptides, to be aggregation prone. It is worth highlighting, however, that full‐length folded S protein did not form amyloid fibrils *per se*, but incubation with proteases, such as the neutrophil elastase, was required to expose amyloidogenic segments [21]. Our study, therefore, demonstrates a novel aggregation behavior of the full‐length S protein in the presence of LPS. Moreover, it has been reported that heparin‐binding sites are common structural features in amyloid proteins, and the interaction with heparin triggers aggregation [22, 23]. S protein contains a receptor‐binding region, which binds to heparin and heparin‐binding proteins [22]. Interestingly, it has been shown that heparin‐binding regions of proteins have an affinity for LPS, an observation providing a further link between these heparin‐binding regions and LPS‐induced amyloid formation [12, 13].

At the molecular level, S protein aggregation has been visualized by cryo‐EM, whereby structures of a COVID‐19 S‐protein‐based vaccine candidate showed the formation of dimers of trimers and trimers of trimers [11]. A short loop nearby the CTD2 of one S protein monomer inserts into the NTD of a monomer from an adjacent S protein, thus creating two points of contact between two S protein trimers. Similar insertions were observed in the trimer of trimers structure; hence, these two points of contact could be the basis of nucleation of higher order oligomers or aggregates. Apart from these two points of contact, our predictions suggest that several regions surrounding the LPS binding pockets on the S1 subunit are also prone to aggregate. It is possible that residues around these regions could act as additional points of contact between neighboring S protein trimers upon LPS binding, thus promoting the formation of even larger aggregates in the presence of LPS. The binding of small hydrophobic molecules, such as haem metabolites, to the LPS binding site on the NTD can induce profound allosteric conformational changes [24]. It is therefore likely that LPS binding can potentially reveal the aggregation‐prone sequences *via* allosteric communication, which subsequently favors larger S‐protein‐LPS aggregates. Our simulations reveal stable lipid A binding to multiple sites on the S protein dimer of trimers and trimer of trimers, suggesting that S‐protein‐LPS complexes can indeed form stable aggregates. Nevertheless, the exact molecular mechanism of how LPS drives the formation of larger S protein aggregates compared to S protein alone, as observed in our electron and fluorescence microscopy experiments, would require further studies.

Our previous research has shown that SARS‐CoV‐2 S protein could act as an additional courier for LPS in the TLR4 pathway, hence resulting in overstimulation and leading to a hyperinflammatory state [2, 5]. This has a significant physiological relevance at ultra‐low levels of LPS (at or below 1 nm) where S protein at nanomolar levels disaggregates LPS micelles and significantly boosts the TLR‐mediated LPS response [2, 25]. The consequences of S protein‐LPS aggregation for potential formation of amyloid aggregates at the high LPS and S protein concentrations used here (50 and 1 μm, respectively), and its relevance *in vivo* clearly needs further investigation. It has been shown that the SARS‐CoV‐2 S protein interacts with heparin and heparan sulfates *via* the RBD and furin cleavage site [26, 27, 28]. Such interactions have been proposed to facilitate aggregation of amyloid proteins in the brain [23]. Additionally, a recent computational study showed that the S protein could also bind to several aggregation‐prone amyloid proteins such as Aβ, α‐synuclein, tau, prions, and TAR DNA binding protein‐43 [29]. It is therefore possible that S protein aggregation, which is enhanced in the presence of a high concentration of LPS, could initiate aggregation of amyloid proteins leading to neurodegeneration in COVID‐19 patients. Interestingly, the herpes simplex virus type 1 whose surface glycoprotein also binds to heparin has been shown to catalyze Aβ_42_ amyloid aggregation *in vitro* and *in vivo* [30]. It is thus imperative to investigate whether LPS under certain conditions may similarly trigger S protein aggregation *in vivo* and subsequent amyloid formation *via* S protein‐LPS interactions with other amyloidogenic proteins. Understanding the link between S protein aggregation and amyloid formation will have important implications in therapeutic interventions of neuropathologies associated with SARS‐CoV‐2 infection.

## Conflict of interest

AS is a founder of in2cure AB, a company developing therapies based on thrombin‐derived host defense peptides. The peptide TCP‐25 and variants are patent‐protected. All other authors declare no competing interests.

## Author contributions

JP and AS conceived the project. JP designed and performed the experiments ThT assay, fluorescence microscopy, electron microscopy, and Blue Native gel assay. FS and PJB designed and performed *in silico* analysis. JP and FS wrote the manuscript with contributions from AS and PJB. All the authors discussed the results and commented on the final manuscript.

## Data Availability

The data that support the findings of this study are available from the corresponding author (jitka.petrlova@med.lu.se) upon reasonable request.
